# Libertinage

**Published:** 1863-10

**Authors:** 


### ART. XII.—LIBERTINAGE.

THE question of libertinage has once more cropped out in the daily press. It is an excellent sign for the future that the medical aspect of this question is now chiefly occupying public attention. It is impossible to dissociate this from the moral aspect, without at the same time cutting from under our feet one of the securest stand-points for effecting good among the unhappy victims to profligacy. If the moral evil be the greatest, it by no means excludes the physical; and the latter touches more narrowly the welfare of the community than is commonly believed. It gives rise to an amount of present suffering, which although originating in most hateful causes, we dare not pass unheeded; and too often, alas! it extends its hideous mischief from father to son, working its evils from generation to generation, poisoning childhood in mind, body, and soul, from the very source. It is the link which binds the physical and mental deterioration of the nethermost classes to that of the highest—which unites in one common bond the degradation of St. James's and St. Giles's. To ignore that terrible cycle which is ever revolving before us, where sin is constantly begetting disease and degradation, and degradation and disease begetting sin, is wilfully and blindly to cast aside the torch which most discloses the hidden roots of the dark evil we would fain eradicate. If we would shatter the cycle, we must strike at all the elements which compose it; but hitherto England has neglected this great truth in her efforts to reclaim the libertine and restrain licentiousness. We must meet libertinage with the hospital as well as the pulpit, if we would hope to make upon t a deeper inroad than we have hitherto done, or stay that poisonous stream which in its insidious flow involves the innocent and the guilty alike.

The great disability occasioned in the army and navy by libertinage is forcing the physical aspect of the question upon the public, at the present moment, in a manner which affords some ground for the belief that it will—to the unspeakable degradation of the soldier and sailor, and detriment to the public service—no longer be left without an attempt at solution. The long indifference of the military authorities and the Government to this evil is a public scandal; the carelessness of the civil authorities is equally a scandal. The public—also apathetic, or at the best but moved to spasmodic attention—have this excuse, that, approaching the subject almost entirely from a moral point of view, they are ignorant of the reality, extent, and degree of physical mischief which the evil involves, and how inextricably this is interlaced with the moral mischief. This ignorance begets a tendency to exaggerate the difficulties which surround the practical treatment of the question, and a vague fear lest, in meddling with the physical evil, however noxious, we should increase the moral. It is felt that whatever the utility of hospitals, that utility must be of the most limited character, unless combined with some measure of police. It is dreaded that if Government should from the public funds make provision for the relief of the evil in the army and navy, it would be offering a premium for vice, and establishing a precedent for the legislative protection of immorality from which public feeling naturally shrinks. We believe that these opinions are erroneous; but it is not the less true that they offer one of the most formidable obstacles to any satisfactory attempt to deal with the evil, and that a preliminary step in our efforts to grapple with it, must be a thorough enlightenment of the public on the sanitary and police aspects of the question by an official inquiry. The evil obtains its worst development under the cloak of, and in consequence of this very dread of interference.

We do not yet know how much of the mischief is created by the neglect to apply existing laws rather than by the need of new ones. Look, for example, at Aldershot. A camp is created apart from any town, and under circumstances where it might be presumed that a little forethought would have secured exemption from the worst mischief of garrison towns. But what occurs? The military authorities give no heed to the subject in the formation of the camp, and take no precautions. The magistrates do not consider that they have any responsibility in the matter, and license public-houses and drinking places indiscriminately. A new village springs up on the borders of the camp, which in an official report on the subject published last year, is described as "inhabited principally by publicans, brothel-keepers, prostitutes, thieves, and receivers of stolen property."* The evil which has thus been permitted to arise grows in magnitude and presently attracts public attention. The sympathy of religious bodies is warmly excited. Missions are formed, churches are built in and near the camp, and many holy men devote their lives to stem the moral mischief. But can it be for a moment believed that if the military and civil authorities had exercised necessary forethought—nay, rightly fulfilled their palpable duties—a large criminal population could have settled down upon the borders of the camp?

But the mischief having been done, let us for a moment observe how it is perpetuated:—

"I visited," says Captain Jackson, "the various places to which the men are generally in the habit of resorting for amusement when off duty. They consist of canteens, public-houses, and beer-houses, nearly all belonging to brewers. The canteens, 18 in number, are the largest, most comfortable, and cheerful huts in the camp. There are 25 public-houses, several of which have large halls attached, fitted up with great splendour. As an inducement to the men to visit these places and spend their money in drink, the attendance of prostitutes, gaily dressed, is encouraged. The proprietors or tenants of these music-halls compete with each other in attempting to make them attractive: first, in their appearance and comfort; secondly, in the varied character of the entertainments; and thirdly, by an excitement of a highly demoralizing and injurious tendency. A small entrance fee is usually charged, but the great remuneration for the very large outlay is derived from the sale of intoxicating drinks. It is well known that the tenants of public-houses in some cases keep prostitutes in their houses, and pass them off as servants, and in other cases provide lodgings for women in the neighbourhood, that they may draw custom to their houses. There are 47 beer-houses, which

---

* *Reports of Captain Jackson, R.A., to the Secretary of State for War, respecting the Present State of Soldiers' Institutes in England.* (Parliamentary paper.) 25th March, 1862.

are, I understand, almost without exception, public brothels of the worst description. The women generally live in these houses and have the free use of certain rooms in consideration for drawing men to drink in the houses, and in some cases the women pay for this privilege, making what they can from the men: they usually treat men well on first arrival, to induce them to return, and rob them when opportunities occur.

"On the 16th July I visited 25 public-houses and beer-houses, and found 80 prostitutes in them, drinking, and in some cases playing cards with soldiers.

"The most craftily-devised plans are in full operation, first to attract, and then to excite and gratify the sensuous passions of the men. One result naturally flowing therefrom is shown in the Statistical Report of the Army Medical Department for the year 1859. The number of admissions to the hospital for diseases incident to lust, at Aldershot, was in excess of the average of all other stations in the United Kingdom.

"Such is the character of the under-current of the camp, so far as the men are concerned, and their mode of spending their leisure time, and such is the character of the places to which they are in the constant habit of resorting. No system could be better imagined or more successfully carried out, if the object were to sap gradually the health of the soldiers, to induce early debility, and to hasten a premature death. The new village of Aldershot, which is inhabited principally by publicans, brothel-keepers, prostitutes, thieves, and receivers of stolen property, has sprung up within a stone's throw of the permanent barracks, which has, unfortunately, been built upon the very borders of the Government property. Hence the men quartered there have the minimum taxation of time and trouble upon frequenting the village, and the maximum of temptation to vice. Five of the worst houses have been put out of bounds by the military authorities. The civil police have confined themselves more particularly to the preservation of external order and decency, and to their other duties respecting life and property. It is a remarkable fact, that no prosecution has been instituted since the formation of the camp against the tenant of any public-house or beer-house for harbouring prostitutes, or keeping a public brothel, or against any prostitute as such. The parish authorities will not incur the expense of prosecuting against the wishes of the ratepayers, a large number of whom, and the most influential, derive pecuniary benefit from the evils in question, and the remainder are naturally indisposed to attempt reforms which would directly affect their neighbours' welfare. They have also pleaded the difficulty of obtaining convictions. I was informed of a most flagrant case in which the police were prepared with indisputable evidence, and had specially retained witnesses; nevertheless, the parish refused to prosecute.

"These evils, which so materially affect the efficiency of the troops, increase the expenditure of the State, and augment the poverty of the country, might be materially lessened if the police were encouraged by the magistrates to collect evidence, and the Treasury were to appoint a prosecutor. This state of things may to a certain extent be inseparable from a camp, but, unfortunately, whatever amount of evil exists, is usually viewed and spoken of as being necessary. I most respectfully submit that such a designation is as false as it is dangerous, and has a tendency to perpetuate the existing amount of the current vices. This amount may be considerably reduced by keeping out of sight everything of an exciting and suggestive character relating to the evils complained of, by lessening the opportunities and increasing the difficulties connected with their indulgence, and by encouraging in the men a taste for useful or harmless occupation of their leisure time."

We do not forget that the principal inhabitants of Aldershot, the parish authorities, and some of the magistrates, protested against this account, and averred that it was a gross exaggeration. Their defence of themselves, however, did not set aside the substantial accuracy of the account, and gave rise to the impression that it was prompted chiefly by the indignant doubt which first possessed them when Captain Jackson's report was made public, than by any sufficient knowledge of the facts debated.

Now there is little question that the action of the authorities in the different garrison towns of the kingdom is the counterpart of that observed at Aldershot, and that the same is largely true of the action of the civil powers concerning this matter in towns generally. We believe that a careful official inquiry into the condition and control of prostitution in large towns would elicit the fact that much of the scandal and disorder to which libertinage gives origin, arises from the absence of any systematic exercise of the powers which the law already gives to the police; and further, that the civil power is held in restraint by the unfortunate tone of public feeling to which we have referred.

Great as are the difficulties which have to be contended with in dealing practically with libertinage (and we have no wish to underrate them), still that they are exaggerated by a want of accurate knowledge of the real stumbling-blocks to efficient interference we have doubt. The difficulties conceived to beset police control are commonly preposterously heightened by inaccurate notions of the extent of the population over which that control is required, and consequent vague fears lest it might trench upon some important political privilege. It is much too little known that prostitutes are, for official purposes, enumerated among the *criminal classes*, and that the police are required to make annual returns of the number of *known* prostitutes in their districts. These returns have been compiled since the establishment of *Judicial Statistics* in 1857. They afford an accurate knowledge of the numbers of those prostitutes who are the source of the great scandals in our public streets, who swell the lists of criminals, and who disseminate disease. An examination of the returns shows that numbers cannot be an insuperable difficulty to regulation, and this does away with one of the commonest objections to interference. We subjoin the return of the prostitute population of England and Wales for the year ending the 29th September, 1862.

The total number of known prostitutes at that date was 29,956—1507 being under sixteen years of age, 28,449 sixteen and above.

Their distribution throughout the country was as follows:—

1. *The Metropolis:*—Including an average radius of 15 miles round Charing Cross, and comprising the district of the Metropolitan Police and the City of London Police: 5795, or 1 in 574·5 population.

2. *Pleasure Towns:*—Brighton, Bath, Dover, Leamington, Gravesend, Scarborough, and Ramsgate: 882, or 1 in 252·3.

3. *Towns depending on Agricultural Districts:*—Ipswich, Exeter, Reading, Shrewsbury, Lincoln, Winchester, Hereford, and Bridgewater: 706, or 1 in 258·6.

4. *Commercial Ports:*—Liverpool, Bristol, Newcastle-on-Tyne, Kingston-on-Hull, Sunderland, Southampton, Swansea, Yarmouth, Tynemouth, and South Shields: 4740, or 1 in 213·7.

5. *Seats of the Cotton and Linen Manufacture:*—Manchester, Preston, Salford, Bolton, Stockport, Oldham, Blackburn, Wigan, Staleybridge, and Ashton-under-Lyne: 1603, or 1 in 524·5.

6. *Seats of the Woollen and Worsted Manufacture:*—Leeds, Bradford, Halifax, Rochdale, Huddersfield, and Kidderminster: 720, or 1 in 609·4.

7.—*Seats of the Small and Mixed Textile Fabrics:*—Norwich, Nottingham, Derby, Macclesfield, Coventry, Newcastle-under-Lyne, and Congleton: 610, or 1 in 485·5.

8. *Seats of the Hardware Manufacture:*—Birmingham, Sheffield, and Wolverhampton: 853, or 1 in 635·6.

*Agricultural Counties.* (a) *Eastern Counties:* Essex, Norfolk, Suffolk, Lincoln: 821, or 1 in 1502·0. (b) *South and South Western Counties:*—Southampton, Wilts, Dorset, Somerset: 1346, or 1 in 806·5. (c) *Midland Counties:*—Cambridge, Bedford, Northampton, Hertford, Oxford, Bucks, Berks: 714, or 1 in 1390·4.

Such is the limit of the prostitute population with which the police would have to deal; and it is as well to remember that it might probably be found, on investigation, that it would be rather by the systematic carrying out and legitimate extension of existing laws and principles, than by the introduction of new, that the hands of the civil powers would be strengthened.

The more strictly medical aspect of the question has been so happily stated in a letter published in the *Times* of the 3rd of September, that we shall make no apology for transferring it entire to our pages.

"To the Editor of the Times.

"Sir,—In your issue of August 31, Captain Coote, R.N., expressed most satisfactorily views which ought to be adopted for the prevention of the most loathsome disease now so prevalent in our naval ports; too much weight cannot be attached to his sentiments, for they have been formed upon actual observation of facts as they exist.

"As one of the medical officers of the Portsmouth Hospital, the one to which he alludes, I may be permitted to make a few remarks. Captain Coote says, 'no power exists whereby women loathsome with disease can be sent to that hospital or can be retained there until cured.' At present no power is needed to force these poor creatures to enter, for our waiting-room is daily thronged with applicants for admission. It is the fear of not obtaining admission which limits the number of patients. But the medical officer needs power to retain a patient until she is cured, or is in such a state that she cannot transmit disease to others. We always observe a restlessness throughout the Lock wards as soon as intelligence enter it that a ship is to be paid off at Spithead; all those who are in a fit condition eagerly seek their discharge, while others (but not all) leave on their own responsibility, with the disease upon them in its most virulent form, and the officers of the hospital are powerless to prevent them; the effects are soon manifest in the wards of the military and naval hospital, which soon teem with victims to the malady; whereas we have the mortification to find before long those who have left us in reality well return for fresh treatment, and those who took their own course come back in a most deplorable condition. To refuse these latter as a punishment is to multiply the evil for which the institution is established. To admit them is our only alternative. Thus have we entailed upon us the labours of Sisyphus—we roll the stone up the hill only for it to roll down again. We are doomed often to see our labours ineffectual and our toil yield no adequate result. It is, beyond doubt, a position which no British charity or sympathy can ameliorate. When an evil is indomitable to such powerful influences, surely it is time for our Legislature to interfere. The argument that it is wrong to license vice cannot here be brought forward. It is merely seeking power to prevent established vice from doing further mischief. Here is a disease the secondary and tertiary effects of which are most destructive to the constitutional stamina of the victim, its hereditary taint pernicious to the offspring, its immediate results debilitating to the sufferer, incapacitating him from labour, yet it is allowed to be at large in our streets, infectious as it is in the highest degree. When a vessel arrives from an unhealthy port it is not thought an infringement upon the liberty of the subject to draw a cordon around her and all her crew and passengers. How can it be thought to be an attack upon the free agency of some abandoned and profligate women to put such restrictions on their liberties as will effectually prevent their disseminating a disease the ravages of which the unborn and innocent infant may yet evince? Such an anomaly in the 19th century will scarcely be credited when the acts of our day shall become matters of history.

"I am writing this because large naval barracks for our seamen are about to be built. These will not tend to improve our condition unless the Government is prepared to adopt some measure to check this monstrous evil. It might be inferred that I deem the Lock wards of our local hospital inert. Far be it from me to say so; upwards of 200 girls are successfully treated in it annually. The history of some I have already alluded to; others the local Penitentiary gains an influence over; I have had the satisfaction of returning some poor girls to their sorrowing and wounded parents, but the supply of the evil still exists, and from that nucleus we must expect a spread of this vile disease.

"Hoping that the publicity you have given to the subject may be the means of awakening the authorities,

"I remain, Sir, your obedient servant,

"J. W. M. Miller, M.D.

"Gloucester-house, Southsea, Sept. 1."

That the great obstacle set forth by Dr. Miller to the usefulness of hospitals is susceptible of being readily removed without interfering unjustly with the liberty of the subject, we have the assurance of distinguished legal authority. It is unquestionably in this direction, and with especial reference to garrison towns and naval stations, that our efforts must first be made. By the regulation of the evil in such towns first, we should obtain the requisite experience for more comprehensive measures. But, to satisfy the prevalent doubts among the public, as well as to ensure that any measures adopted are duly matured and based upon a just knowledge of the mischiefs to be remedied, an official investigation should precede any legislative or official action. The experience of India teaches us the necessity for this, so far as it affects the adoption of efficient measures. There hospitals entirely failed to give the relief sought from them, and they fell into disuse. But the evidence laid before the recent Royal Commission on the Sanitary State of the Army in India indicates that the failure arose chiefly from the fact that more was expected from these establishments than they could possibly perform; and thus an error of conception led to dissatisfaction, distrust, and finally, loss of confidence in their usefulness. The Commission recommend the re-establishment of special hospitals, as well as of the systematic police regulation necessary to enforce their utility; but it is unmistakeably manifest in their Report that the evidence laid before the Commission failed in that precision and completeness which was requisite to give their recommendation the force it should possess.

It is fortunate that we possess in Mr. Acton's* able works on "Prostitution" and the "Reproductive Organs,"† trustworthy guidance in the principles which should govern us in dealing with the medical aspects of libertinage. What we require in addition is a more thorough knowledge of the circumstances which should direct the application of these principles to this country; and this, we hold, is to be acquired solely by a Government inquiry into the subject.

* *Prostitution, considered in its Moral, Social, and Sanitary Aspects, in London and other large Cities. With Proposals for the Mitigation and Prevention of its attendant Evils.* By Wm. Acton, M.R.C.S. London, 1857.

† *The Functions and Disorders of the Reproductive Organs in Childhood, Youth, Adult Age, and Advanced Life, considered in their Physiological, Social, and Moral Relations.* By the same Author. London, 1862.

